# Dendrobium nobile Lindl. Alkaloids Decreases the Level of Intracellular β-Amyloid by Improving Impaired Autolysosomal Proteolysis in APP/PS1 Mice

**DOI:** 10.3389/fphar.2018.01479

**Published:** 2018-12-18

**Authors:** Jing Nie, Lin-Shan Jiang, Yu Zhang, Yong Tian, Li-Sheng Li, Yan-Liu Lu, Wen-Jin Yang, Jing-Shan Shi

**Affiliations:** Key Laboratory of Basic Pharmacology of Ministry of Education and Joint International Research Laboratory of Ethnomedicine of Ministry of Education, Zunyi Medical University, Zunyi, China

**Keywords:** Dendrobium nobile Lindl. alkaloids, alzheimer’s disease, β-amyloid peptide, macroautophagy, lysosomal acidification

## Abstract

As the major degradation pathway for long-lived proteins and organelles, macroautophagy is a decisive factor for the survival and longevity of cells. The existing evidence indicates that the disruption of substrate proteolysis in autolysosomes is the main mechanism underlying autophagy failure in Alzheimer’s disease (AD). Thus, the restoration of normal lysosomal proteolysis and autophagy efficiency is a novel therapeutic strategy in the treatment of AD. In this study, 9-month-old APPswe/PS1ΔE9 transgenic (APP/PS1) mice were administered Dendrobium nobile Lindl. alkaloids (DNLA, 40 and 80 mg/kg) or Metformin (80 mg/kg), and age-matched wild-type mice were administered an isovolumic vehicle orally once a day for 4 months. The results demonstrated that DNLA significantly improved learning and memory function in APP/PS1 transgenic mice in the Morris water maze. Furthermore, DNLA could increase the expression of the v-ATPase A1 subunit to facilitate lysosomal acidification, prompt the dissociation of the cation independent-mannose-phosphate receptor from cathepsin (cat) D, promote the proteolytic maturation of cat D, increase the degradation of accumulated autophagic vacuoles (AVs) and β-amyloid (Aβ) contained in the AVs, and alleviate neuronal and synaptic injury. These findings demonstrate that DNLA improves learning and memory function in APP/PS1 mice, and the mechanisms appear to be due to the promotion of intracellular Aβ degradation by increasing the protein level of v-ATPase A1 and then improving autolysosomal acidification and proteolysis.

## Introduction

Alzheimer’s disease (AD) is a common age-related neurodegenerative disorder characterized by extracellular senile plaques, intracellular neurofibrillary tangles, and neuronal degeneration along with significant synaptic loss (Selkoe, 2002; Cavallucci et al., 2012). Although the precise molecular mechanism underlying the pathogenesis of AD has yet to be fully elucidated, it is widely accepted that the β-amyloid (Aβ) peptide cascade plays a critical role in the development of AD (Jin et al., 2014). The imbalance between the generation and clearance of Aβ leads to increased Aβ levels in the central nervous system (CNS) (Zolezzi et al., 2014). Accumulation of Aβ initiates a cascade of events, such as activation of astrocytes and microglia, initiation of inflammatory responses, alteration of neuronal ionic homeostasis etc., resulting in neuronal/synaptic dysfunction and loss, and causing patients to exhibit the symptoms of dementia (Klafki et al., 2006; Mawuenyega et al., 2010). Modulation of the pathological progression of the Aβ peptide is a key strategy to slow down AD progression.

A comparison of the generation and clearance of CNS Aβ in patients with symptomatic AD and in normal individuals indicated that the average production rates of Aβ_1-40_ and Aβ_1-42_ did not differ between the control group and the AD group. However, the mean clearance rates of Aβ_1-40_ and Aβ_1-42_ were slower in the AD group than in the control group, suggesting a more important role of impaired Aβ clearance in the development of AD (Mawuenyega et al., 2010). It is believed that Aβ is generated at several locations in neurons, including the Golgi complex, endosomes, and endoplasmic reticulum (Cataldo et al., 2004). It was demonstrated that Aβ was generated in autophagic vacuoles (AVs), which abnormally aggregated in the affected neurons during macroautophagy (Yu et al., 2005). As the major degradative pathway for organelles and long-lived proteins, autophagy is essential for the survival of neurons (Nixon and Yang, 2011). At low levels of macroautophagy induction, Aβ generated in AVs is soon degraded by lysosomes. However, current evidence indicates that substrate proteolysis within autolysosomes is damaged in the brains of AD patients, resulting in the massive accumulation of incompletely digested substrates including Aβ contained in AVs (Nixon and Yang, 2011). Abnormal degradation of AVs may be a major reason for the reduction of Aβ clearance, suggesting that restoring normal lysosomal proteolysis and autophagy efficiency may promote the clearance of intracellular Aβ, and indicating the potential of autophagy modulation as a therapeutic strategy.

Our previous studies indicated that Dendrobium nobile Lindl. alkaloids (DNLA), which was originally extracted from the traditional Chinese herbal medicine *Dendrobium nobile*, can improve the neuronal disruption caused by lipopolysaccharide (Li et al., 2011), and oxygen-glucose deprivation and reperfusion (Wang et al., 2010), and decrease neuronal apoptosis, hyperphosphorylation of tau protein (Shu et al., 2013), and Aβ deposition in the rat brain (Chen et al., 2008). Furthermore, in *in vitro* experiments, we found that DNLA could alleviate Aβ_25-35_-induced axonal injury by improving autophagic flux in neurons (Li et al., 2017). The present study aims to investigate the effect of DNLA on improving learning and memory ability in amyloid precursor protein/presenilin 1 (APP/PS1) mice, and further explore the mechanism underlying the regulation of the autophagic pathway.

## Materials and Methods

### Reagents and Antibodies

Dendrobium was purchased from Xintian Traditional Chinese Medicine Industry Development Co., LTD., of Guizhou Province. DNLA was isolated from the extracts, and analyzed by LC MS/MS. Alkaloids accounted for 79.8% of the DNLA, and mainly contained 92.6% dendrobine (C_16_H_25_O_2_N), 3.3% dendrobine-*N*-oxide (C_16_H_25_O_3_N), 2.0% nobilonine (C_17_H_27_O_3_N), 0.9% dendroxine (C_17_H_25_O_3_N), 0.32% 6-hydroxy-nobilonine (C_17_H_27_O_4_N), and 0.07% 13-hydroxy-14-oxodendrobine (C_16_H_23_O_4_N) (Nie et al., 2016). Adeno-associated vector (AAV) 9-monomeric red fluorescent protein (mRFP)-green fluorescent protein (GFP)-light chain 3 (LC3) was purchased from Hanbio Biotechnology Co., Ltd., Anti-Aβ_1-40_ (ab12265), anti-Aβ_1-42_ (ab10148), anti-lysosomal-associated membrane protein 2 (LAMP2) (ab13524), anti-cathepsin (cat) D (ab6313), anti-beclin1 (ab55878), anti-mannose-6-phosphate receptor (M6PR) [cation independent (CI)] (ab124767), and donkey anti-rabbit-IgG H&L (Alexa Fluor 488) were purchased from Abcam (Cambridge, United Kingdom). Anti-P70s6 kinase (9202), anti-phosphor-p70s6 kinase (Thr389) (9206), and anti-LC3A/B(D3U4C)XP (12471) were purchased from Cell Signaling Technology (Boston, MA, United States). Anti-v-ATPase A1(H-140) (sc-28801) was purchased from Santa Cruz Biotechnology (Santa Cruz, CA, United States). Rhodamine (TRITC)-conjugated goat anti-rat IgG (H+L) (SA00007-7) and rhodamine (TRITC)-conjugated goat anti-mouse IgG (H+L) (SA00007-1) were purchased from Proteintech Group (Chicago, IL, United States).

### Animals

Male APPswe/PS1ΔE9 transgenic (APP/PS1) mice and their wild-type (WT) littermates (Certificate no: SCXK 2010-0001) were purchased from the Model Animal Research Centre of Nanjing University (China). The mice were housed in SPF-grade animal facilities (Certificate no.: SYXK 2014-003) at 22–23°C with a 12 h light/dark cycle. Mice were provided *ad libitum* access to food and water until they reached 13 months of age. All animal procedures were approved by the Animal Experimentation Ethics Committee of Zunyi Medical University.

### Experimental Design

Male 9-month-old APP/PS1 mice were randomly divided into four groups: the DNLA groups (40 and 80 mg/kg), metformin (Met) (80 mg/kg) group (Łabuzek et al., 2010; Campbell et al., 2017), and APP/PS1 control group (*n* = 10 each). Age-matched WT male mice comprised the WT control group (*n* = 10). Once daily for 4 months, DNLA and Met were administered to the DNLA and Met groups, and an isochoric vehicle was administered to the APP/PS1 and WT control groups.

### Morris Water Maze Experiments

Four months after the administration of DNLA, spatial learning and memory function were measured by the Morris water maze (MWM). The MWM experiment was conducted in a 120 cm white pool with a 10 cm escape platform placed 1 cm below the water surface in the center of the target quadrant, and data were recorded and analyzed by the TopView Animal Behavior Analyzing System (Version 3.00). The MWM is divided into two steps, and the place navigation test is the first step. Mice were released from one of three quadrants without platforms. Each trail lasted for 60 s or ended as soon as the mouse climbed onto the platform. The time required for the mice to climb onto the platform within 60 s was recorded as the escape latency. We regarded the time required as 60 s if the mouse failed to find the platform within 60 s. The spatial probe test was the second step and was performed on day 5. The platform was removed and each mouse was allowed to swim for 60 s in the pool. Simultaneously, the searching distance, searching time, swimming speed, and frequency crossing the target quadrant were measured (Edwards et al., 2014; Milner et al., 2014).

### Intracerebroventricular Injection of AAV9-mRFP-GFP-LC3

Two mice from each group were anesthetized with 7% chloral hydrate (35–45 mg/kg, i.p.) and fixed in a stereotaxic instrument (RWD Life Science, China). AAV9-mRFP-GFP-LC3 (3 μl, 10^12^v.p) was then injected into the lateral cerebral ventricles of each mouse via a 5 μl microinjector (Castillo et al., 2013). The injection site was posterior to the bregma = -0.4 mm, mediolateral to the midline = 1.2 mm, and dorsoventral to the skull = 2.7 mm. At 4 weeks post-vector administration, the animals were euthanized and the brains were analyzed by fluorescence microscopy.

### Hematoxylin and Eosin Staining

After the MWM test, three mice were randomly selected from each group anesthetized with 7% chloral hydrate, and then perfused with phosphate-buffered saline (PBS) (0.1 M, 4°C) via the ascending aorta, followed by 4% paraformaldehyde until the tail and limbs were rigid. Thereafter, the brains were removed and bisected. Half of the brain was fixed with 4% paraformaldehyde for 7 days and cut into coronal sections (4 μm thick) for hematoxylin and eosin (H&E) staining. The other half of the brain was subjected to gradient dehydration in 20 and 30% glucose solution and cut into coronal sections (30 μm thick) for immunofluorescence staining.

### Immunofluorescence Staining

The brain slices (30 μm thick) were washed three times with PBS to remove the cryoprotectant, dipped in 0.3% Triton-X-100 for 15 min, and then blocked in goat serum for 30 min. After washing again with PBS, the slices were treated with the appropriate primary antibodies diluted in the blocking solution at 4°C overnight. The antibodies used were as follows: rabbit anti-LC3 (1:250), anti-Aβ_42_ (1:100), anti-v-ATPase (1:50), and anti-M6PR (1:100); rat anti-LAMP2 (1:100); and mouse anti-cat D (1:100). The slices were incubated with donkey anti-rabbit Alexa 488 (1:1000), goat anti-rat IgG (H+L) (1:100), and goat anti-mouse IgG (H+L) (1:100) for 1 h at 37°C. After washing with PBS, the slices were mounted on glass slides. Images were acquired using an epifluorescence microscope (Olympus) (Kim et al., 2014; Wang et al., 2017).

### Western Blot Analysis

Five mice from each group were euthanized after the MWM test, and the hippocampal tissues of half of the brains were collected and homogenized in radioimmunoprecipitation assay lysis buffer (1:5, w/v). Protein concentrations were determined by a bicinchoninic acid protein assay. A total of 45 μg of protein was applied. After electrophoresis, the bands were transferred onto polyvinylidene difluoride membranes. The membranes were blocked with 5% nonfat dry milk in tris-buffered saline with Tween 20 buffer for 1 h at 22 ± 2°C, then incubated with a primary antibody: anti-Aβ_1-40_ (1:1000), anti-Aβ_1-42_ (1:1000), LC3A/B (1:1000), beclin1 (1:1000), p70S6 kinase (p70S6K) (1:1000), Pp70s6k (1:1000), cat D (1:1000), cat B (1:1000), or v-ATP (1:500) at 4°C overnight. The following day, after incubation with horseradish peroxidase-labeled goat anti-rabbit IgG (Beyotime Biotechnology, China; A0208, 1:2000) or goat anti-mouse IgG (1:5000) at 22 ± 2°C for 1 h. The blots were visualized using an enhanced chemiluminescence western blot detection kit (7Sea Biotech, China) and scanned with the Gel Imaging system. The band intensity was quantified using Quantity One 1-D analysis software v4.52 (BioRad, Hercules, CA, United States).

### Electron Microscopy

Half of the brain was used for western blot detection, and the other half was fixed with 2.5% glutaraldehyde for electron microscope detection. The detection was completed in the Central Laboratory of Army Medical University in Chongqin City. The brains then underwent fixation in 1% osmic acid at 4°C for 3 h, gradient acetone dehydration, a 1:1 embedding liquid and acetone soak at 22 ± 2°C for 3 h, 100% embedding fluid soak at 37°C for 2 h, embedding at 35°C overnight, polymerization at 45°C for 12 h and 60°C for 48 h, then incubated in the dryer and sliced by a Leica EMUC7 ultrathin slicer at a thickness 60–70 nm. The slices were observed under the electron microscope after double staining with lead citrate. Three randomly selected electron microscopy images per animal were captured and the number of synapses in each captured field was counted.

### Statistical Analysis

All data are presented as the mean ± standard error. Data were analyzed by SPSS 20.0 statistics software by a one-way ANOVA or student’s *t*-test. *P* < 0.05 was considered to be statistically significant.

## Results

### DNLA Improved Spatial Learning and Memory Impairment in APP/PS1 Mice

**FIGURE 1:**
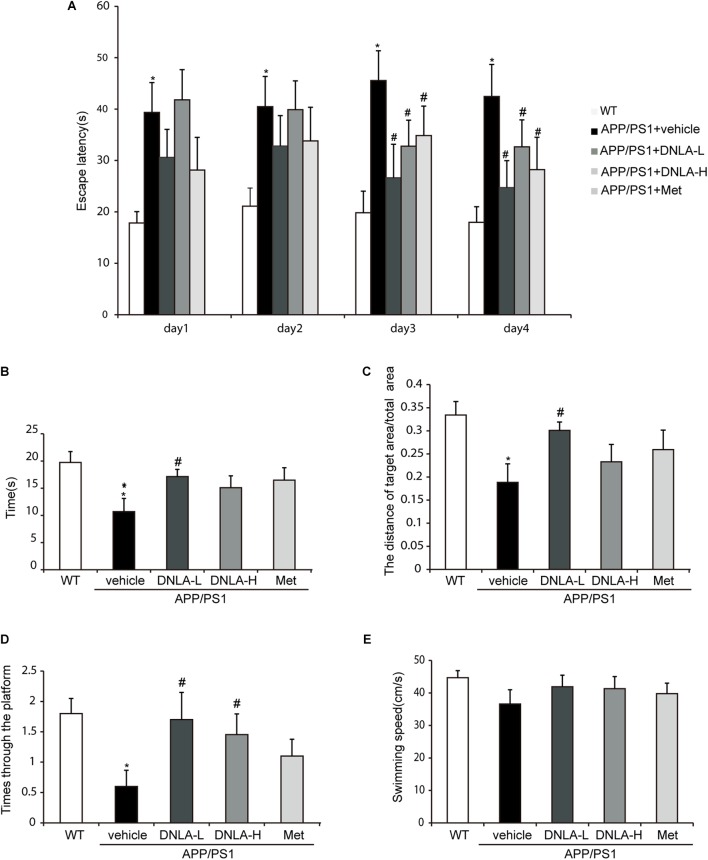
Dendrobium nobile Lindl. alkaloids (DNLA) improved the learning and memory ability of 13-month-old APP/PS1 mice. Nine-month-old APP/PS1 transgenic mice were fed with DNLA (40 and 80 mg/kg) and metformin (Met) (80 mg/kg), while the APP/PS1 control group and WT control group were fed with distilled water orally once a day for 4 months. **(A)** The escape latency of the mice reached the hidden platform from day 1 to day 4. **(B)** The time spent in the target quadrant. **(C)** The adjusted searching distance in the space probe test. **(D)** The frequency crossing the target quadrant. **(E)** The average swimming speed. Data were presented as mean ± S.E.M. (*n* = 10). ^∗^*p* < 0.05, vs. WT group, ^#^*p* < 0.05 vs. APP/PS1group.

In the place navigation test, from the first day, the model group exhibited extended escape latencies. Treatment with DNLA and Met significantly reduced the escape latencies on the third and fourth days (Figure 1A). In the spatial probe test, the exploring time and distances in the target quadrant, the frequency of crossing the target quadrant, and the swimming speeds were recorded. The swimming speeds suggested that there were no significant differences among these groups (Figure 1E). The model group exhibited a decrease in the mean exploring time and distance (Figures 1B,C), as well as in the frequency of crossing the target quadrant (Figure 1D). Furthermore, 40 mg/kg DNLA treatment significantly increased the mean exploring time and distance and the frequency of crossing the target quadrant. Taken together, these data indicated that DNLA could improve learning and memory impairment in APP/PS1 mice.

### DNLA Alleviated the Neuronal Injury in the Hippocampi of APP/PS1 Mice

**FIGURE 2:**
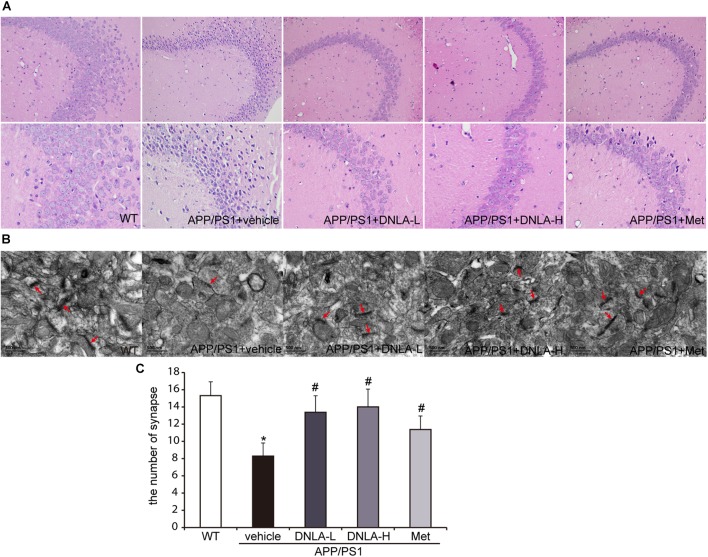
Dendrobium nobile Lindl. alkaloids (DNLA) alleviated the neuronal injury of hippocampi in 13-month-old APP/PS1 mice. **(A)** The sections of the hippocampal CA3 region were obtained and stained with HE (magnification 200×, 400×). **(B)** The neuronal synapses in hippocampus were observed under electron microscope (50kx×). **(C)** The number of synapses in hippocampus. Data were presented as mean ± S.E.M. (*n* = 5). ^∗^*p* < 0.05 vs. WT group, ^#^*p* < 0.05 vs. APP/PS1 group.

Mounting evidence has indicated that dementia is attributed to synaptic dysfunction and neuronal degeneration (Ryan et al., 2010). In the present study, H&E staining revealed that the neuron staining was abnormal with regard to the nuclear condensation, and the number of synapses was decreased in the model group. These neuronal pathological alterations in the model group may represent a molecular basis underlying the learning and memory impairment in APP/PS1 mice. DNLA treatment significantly decreased the number of abnormally stained neurons and increased the number of synapses (Figure 2).

### DNLA Decreased the Expression of Intracellular Aβ_1-42_ in the Hippocampi of APP/PS1 Mice

**FIGURE 3:**
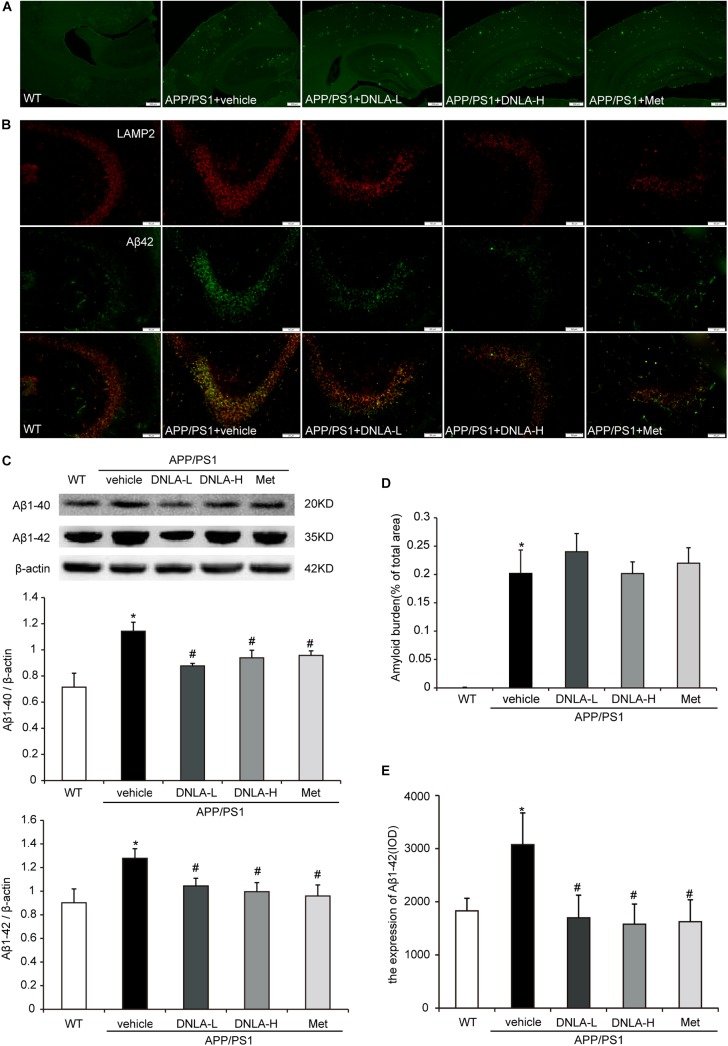
Dendrobium nobile Lindl. alkaloids (DNLA) decreased the expression of intracellular Aβ_1-42_ of hippocampi in 13-month-old APP/PS1 mice. **(A)** The sections of the hippocampi were stained with fluorescent dye Thioflavin-T. **(B)** Hippocampal tissue was analyzed by double- immunofluorescence through LAMP2 and Aβ_1-42_. **(C)** Immunoblot analyses of Aβ_1-42_ and Aβ_1-40_. Data were presented as mean ± S.E.M. (*n* = 5). ^∗^*p* < 0.05 vs. WT group, ^#^*p* < 0.05 vs. APP/PS1 group. **(D)** Quantification of amyloid fiber plaques described in **A**. Data were presented as mean ± S.E.M. (*n* = 3). **(E)** Quantification of intracellular Aβ42 described in B. Data were presented as mean ± S.E.M. (*n* = 3). ^∗^*p* < 0.05 vs. WT group, ^#^*p* < 0.05 vs. APP/PS1 group.

As mentioned above, it has been indicated that dementia is attributed to synaptic dysfunction and neuronal loss in the hippocampus and its associated cortex, which are caused by the accumulation of Aβ oligomers. We therefore examined the extracellular amyloid peptide fibrils in the hippocampal tissue using the fluorescent dye, Thioflavin-T, a benzothiazole dye that exhibits increased fluorescence upon binding to amyloid fibrils (Khurana et al., 2005). As indicated in Figure 2, many amyloid peptide fibrils were found to be deposited in the cortex and hippocampus in APP/PS1 mice. Administration of DNLA and Met for 4 months did not reduce the number of amyloid fibrils in the brains of APP/PS1 mice. However, western blot analysis of Aβ_1-42_ and Aβ_1-40_ indicated that DNLA significantly reduced the Aβ_1-42_ and Aβ_1-40_ protein levels in the hippocampi of APP/PS1 mice. We further examined the intracellular Aβ42 expression via immunofluorescence labeled with anti-Aβ_1-42_, and found that the intracellular Aβ_1-42_ expression was significantly reduced in the DNLA groups. Moreover, double-immunofluorescence labeled with Aβ_1-42_ and LAMP2 antibodies confirmed that Aβ_1-42_ was colocalized with LAMP2 (Figure 3).

### DNLA Decreased the Number of AVs in the Hippocampi of APP/PS1 Mice

**FIGURE 4:**
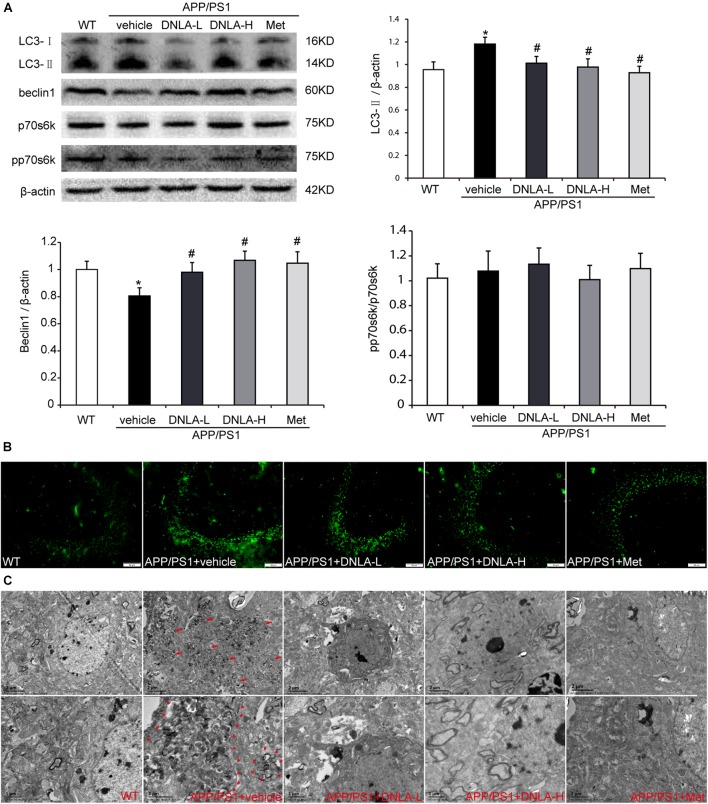
Dendrobium nobile Lindl. alkaloids (DNLA) decreased the AVs in hippocampi of 13-month-old APP/PS1 mice. **(A)** Hippocampal tissue was subjected to western blotting analysis. Data are presented as mean ± S.E.M. (*n* = 5). ^∗^*p* < 0.05 vs. WT group, ^#^*p* < 0.05 vs. APP/PS1 group. **(B)** Immunofluorescence labeled with LC3B antibodies. **(C)** The AVs in hippocampus were observed under electron microscope (12kx×, 20kx×).

It has been reported that AVs within the brain are a major reservoir of intracellular Aβ (Yu et al., 2005). Therefore, we assumed that the number of AVs also increased in the hippocampi of APP/PS1 mice. In order to examine autophagosome formation, we evaluated the expression of LC3-II in the hippocampus by western blot analysis and found that the LC3-II level was higher in the APP/PS1 control group than in the WT group. Consistent with the western blot findings, the results of immunofluorescence test also indicated there were more LC3-positive vesicles in the APP/PS1 mice than in the WT mice. Furthermore, we also observed significant accumulation of AVs in the hippocampi of the APP/PS1 mice under the electron microscope, as indicated in Figure 4. These findings indicated that DNLA could decrease the number of AVs in the hippocampi of APP/PS1 mice.

### DNLA Had No Significant Effect on the Phosphorylation of p70S6K in the Hippocampi of APP/PS1 Mice

The accumulation of AVs in cells may be due to the enhancement of autophagic induction, or may be caused by delayed degradation of the AVs (Rubinsztein et al., 2005). We further assessed the mechanistic target of rapamycin (mTOR)-mediated induction of autophagy by measuring the phosphorylation state of p70S6K. When the mTOR was suppressed, p70S6K phosphorylation decreased, and autophagy was induced. Neither the levels of total p70S6K nor its phosphor-epitope (Thr389) as evaluated by quantitative immunoblotting analyses exhibited any differences between the WT group and the APP/PS1 control group. In addition, there were no significant differences between the APP/PS1 control group and the DNLA group, suggesting that the accumulation of AVs is not due to the increased autophagic activity in the aged APP/PS1 mice, and DNLA did not significantly induce autophagy in the 13-month-old APP/PS1 mice (Figure 4). In addition, as indicated in Figure 4, it was found that the protein expression of beclin1, a “core” element in autophagosome membrane formation (Zhou et al., 2015) was decreased in the APP/PS1 group. DNLA increased the expression of beclin1 (Figure 4).

### DNLA Improved the Clearance of Autolysosomes in the Hippocampi of APP/PS1 Mice

**FIGURE 5:**
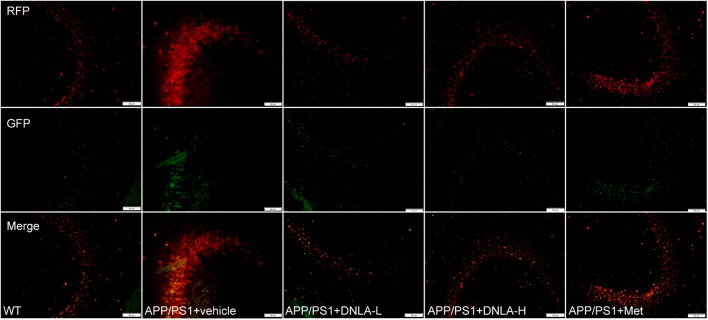
Dendrobium nobile Lindl. alkaloids (DNLA) improved clearance of autolysosomes in hippocampi of 13-month-old APP/PS1 mice. The APP/PS1 transgenic mice of 9 months old were fed with DNLA (40 and 80 mg/kg) and metformin (Met) (80 mg/kg). In contrast, APP/PS1 control group and WT control group were fed with isovolumic vehicle per day for 4 months. One month before the end of administration, Lateral ventricle was injected with AVV9_mRFP-GFP-LC3B. The brain section was observed under the fluorescence microscope.

Since the autophagic pathway of the 13-month-old APP/PS1 mice was not apparently induced, the accumulation of AVs may be related to the degradation process. In the present study, the dynamic autophagy flux reporter, RFP-GFP-LC3, was introduced and expressed in the CNS via the lateral ventricular injection of AAV-mediated delivery (Castillo et al., 2013). This method is based on the sensitivity of GFP fluorescence signal to the acidic environment in lysosomal cavity, resulting in inactivation. Thus, colocalization of GFP and mRFP fluorescence (45yellow puncta) indicates that the tandem protein is not localized in compartments fused with a lysosome (i.e., on the phagophore or within the autophagosome). In contrast, detection of the mRFP signal without GFP (red puncta), indicates that the protein is located in the autolysosome (Matus et al., 2014). We found that the green LC3 puncta were significantly reduced compared to the mRFP puncta in the hippocampi of all mouse groups, indicating efficient autophagosome-lysosome fusion. However, compared to that in WT mice, the red LC3 puncta significantly increased in the hippocampi of APP/PS1 mice. These results suggested that autolysosome degradation was impaired in 13-month-old APP/PS1 mice. After administration of DNLA, the red LC3 puncta were significantly decreased, suggesting that DNLA could improve autolysosome clearance in the hippocampus (Figure 5).

### DNLA Improved Cat D Maturation in the Hippocampi of APP/PS1 Mice

**FIGURE 6:**
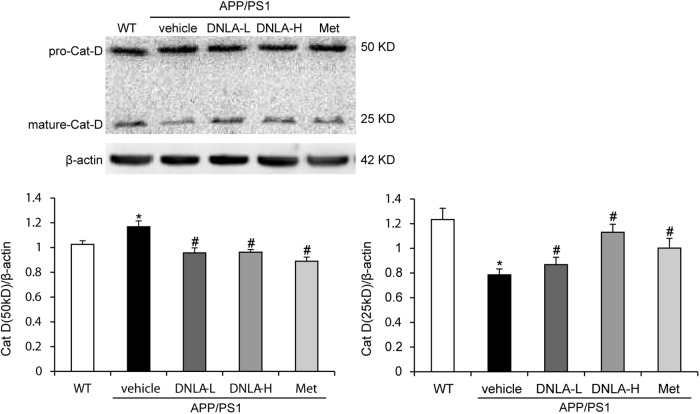
Dendrobium nobile Lindl. alkaloids (DNLA) improved cathepsin D maturation in hippocampi of 13-month-old APP/PS1 mice. Data are presented as mean ± S.E.M. (*n* = 5). ^∗^*p* < 0.05 vs. WT group, ^#^*p* < 0.05 vs. APP/PS1 group.

After the fusion of autophagosomes and lysosomes, the autophagosomes are degraded by the hydrolytic enzymes in the lysosomes. We examined the protein expression of cat D. Cat D is the major aspartic protease of lysosomes, and is synthesized as a pre-proenzyme that is removed from the signal peptide that is targeted to endosomes or lysosomes. In the endosomes, proteolytic removal of the N-terminal propeptide leads to the 48 kDa intermediate form, which is processed to yield two final domain mature enzymes consisting of a heavy chain and a light chain that are non-covalently linked (Stoka et al., 2016). Western blot analysis of‘ cat D revealed lower expression of the mature single chain enzyme in APP/PS1 mice than in WT mice (50 kDa), while an increase in the proteolytic generation of the 25 kDa forms of the mature enzyme was found in the model mice. As indicated in Figure 6, DNLA significantly increased the expression of mature cat D and decreased the premature cat D level, indicating that DNLA could promote the maturation of cat D, and thus enhance the proteolytic activity of cat D (Figure 6).

### DNLA Improved Lysosome Acidification in the Hippocampi of APP/PS1 Mice

**FIGURE 7:**
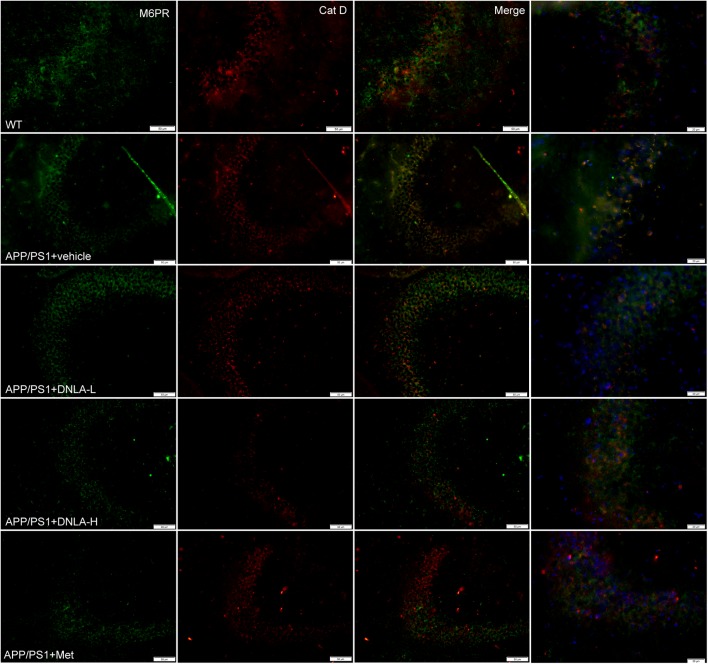
Dendrobium nobile Lindl. alkaloids (DNLA) improved lysosome acidification in hippocampus of 13-month-old APP/PS1 mice. Double-immunofluorescence utilizing M6PR and Cat D antibodies.

No endogenous inhibitors of cat D are known and the major factor in regulating mature cat D activity seems to be the pH (Stoka et al., 2016). The cat D maturation was impaired in the hippocampi of 13-month-old APP/PS1 mice indicated that the lysosomal pH had increased. We evaluated the degree of acidification in lysosomes by evaluating another process that requires an acidic lysosomal environment. All cathepsins are targeted to lysosomes mainly via the M6PR pathway. The M6PR combines with cat D in an environment of a pH value of 6-7; during this process, the pH value drops to 5.3–5.5. The MPR has low affinity for cat D. Cat D combined with the M6PR can be located in the Golgi complex or cytomembrane and is transported to lysosomes, but the normal pH value in the lysosomes is <5 (Stoka et al., 2016). Therefore, the M6PR dissociates from cat D and returns to the Golgi complex or cytomembrane, thus completing the *trans*-shipment of cathepsins. By utilizing double immunofluorescence labeled with antibodies against cat D and the CI-MPR, it was found that most of the cat D-positive vesicles were CI-MPR negative in the WT group. However, nearly all of the vesicles were CI-MPR positive in the APP/PS1 control group, indicating that the dissociation of the CI-MPR from cat D was damaged in APP/PS1 mice, and the pH value in lysosomes was increased. However, it was found the cat D-positive vesicles were CI-MPR negative in the DNLA and Met groups, suggesting that DNLA and Met could acidify lysosomes (Figure 7).

### DNLA Increased the Expression of v-ATPase A1 in the Hippocampi of APP/PS1 Mice

**FIGURE 8:**
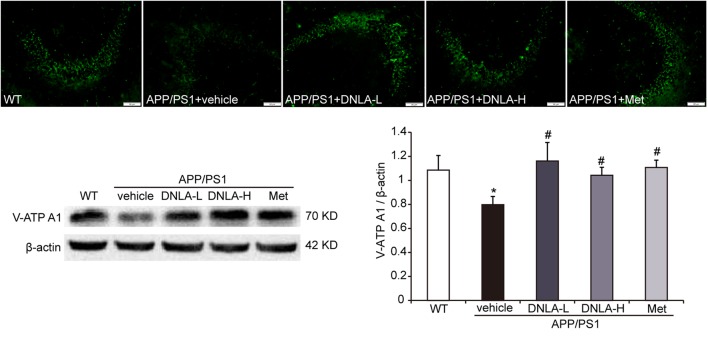
Dendrobium nobile Lindl. alkaloids (DNLA) increased the expression of v-ATPaseA1 in hippocampi of 13-month-old. Data are presented as mean ± S.E.M. (*n* = 5).^∗^*p* < 0.05 vs. WT group, ^#^*p* < 0.05 vs. APP/PS1 group.

v-ATPase is a proton pump through which the acidic environment of lysosomes and other membrane-bound compartments are established and maintained by pumping protons into the lumen (Mauvezin et al., 2015). Western blot analysis and immunofluorescence indicated that the v-ATPase A1 protein expression was significantly decreased in APP/PS1 mice. The v-ATPase A1 expression in the hippocampi of APP/PS1 mice was remarkably reversed by DNLA and Met, suggesting that DNLA could improve lysosome acidification by increasing the expression of v-ATPase A1 (Figure 8).

## Discussion

The present study clearly demonstrated that 4 month treatment of DNLA ameliorated the AD pathology in aged APP/PS1 transgenic mice, as evidenced by behavioral tests, morphology, and reduced Aβ deposition in the hippocampus. More importantly, we observed autophagy as a protective effect of DNLA, including increased v-ATP A1, reduced lysosomal pH, and enhanced CI-MPR dissociation from cat D for proteolysis, thus increased degradation of Aβ-containing AVs.

Alzheimer’s disease is a complex heterogeneous disease, the etiology and pathogenesis of which are still unclear (Zolezzi et al., 2014). The APP, PS1, and PS2 genes have been implicated in AD. Crossing APP mutant mice with mice expressing mutant *PS1* genes resulted in the dramatic development of amyloid pathology and memory deficits (Borchelt et al., 1997). The present study used aged APP/PS1 transgenic mice to determine if the therapeutic effects of DNLA on learning and memory deficits are novel. Our results indicated that DNLA improved learning and memory disorders in APP/PS1 mice, as evidenced by the decreased escape latency and longer exploration time in the place navigation test, as well as the exploration distance and the frequency of crossing the target quadrant in the spatial probe test. In addition, H&E staining revealed that the neuron staining was abnormal with regard to nuclear condensation, and the number of synapses was decreased in the model group. DNLA treatment significantly decreased the number of abnormally stained neurons and increased the number of synapses. These results demonstrated the protective effects of DNLA treatment in APP/PS1 transgenic mice.

The classical amyloid cascade hypothesis supports the notion that increased Aβ production and extracellular accumulation leads to progressive synaptic and neuronal injury resulting in wide-spread neuronal dysfunction and dementia (Cavallucci et al., 2012). However, there is growing evidence that early intraneuronal accumulation of Aβ peptides is one of the key events leading to synaptic and neuronal dysfunction (Bayer and Oliver, 2010), while plaque pathology has a weaker impact on neurodegeneration. In this study, we found that the protein levels of Aβ_1-40_ and Aβ_1-42_ significantly increased in the hippocampi of 13-month-old APP/PS1 mice, and observed an increase in the amount of extracellular amyloid plaques and intracellular Aβ_42_. Treatment with DNLAs for 4 months resulted in a significant decrease in the protein expression of Aβ, but the amyloid plaques exhibited no apparent change. Furthermore, immunofluorescence staining with anti-Aβ_42_ indicated a decrease in the amount of intracellular Aβ_42_, suggesting that the reason underlying the DNLA-induced alleviation of neuron and synaptic injury may be related to the reduction in the amount of intracellular Aβ. We also found that a small amount of amyloid plaques began to appear in 7-month-old APP/PS1 mice by thioflavin-T staining. At the age of 9 months, the number increased significantly and typical pathological changes of AD appeared, but the extracellular amyloid deposition in the 13-month-old APP/PS1 mice was not significantly increased compared with that at 9 months (Supplementary Figure S1). Thus, we consider the possibility that DNLA has no reversal effect on the formed amyloid plaques.

The aggregation of Aβ in neurons is closely related to the balance between Aβ secretion and the metabolism of Aβ in the cell. Aβ is believed to be generated at several sites within neurons, including endosomes, the Golgi complex, and the endoplasmic reticulum (Cataldo et al., 2004). Yu et al. (2005) reported that AVs in the brain are a major reservoir of intracellular Aβ; Aβ is generated in AVs during macroautophagy and is subsequently degraded within lysosomes. In this study, the western blot and electron microscopy results indicated that the number of AVs increased in 13-month-old APP/PS1 mice, similar to the increase in the amount of Aβ.

Autophagy is the major degradative pathway for organelles and long-lived proteins, which is essential for the survival of neurons. Autophagy constantly occurs in neurons, and efficient clearance of autophagosomes by lysosomal degradation makes AVs uncommon in neurons of the healthy brain (Boland et al., 2008). Mounting evidence has implicated defective autophagy in the pathogenesis of AD; the progressive disruption of autophagy leads to the accumulation of AVs, many containing Aβ, in dystrophic neuritis in the AD brain (Nixon and Yang, 2011). Conditions that either stimulate AV production or delay or impair maturation of AVs to lysosomes might be expected to increase the number of AVs and raise intracellular Aβ levels. We first evaluated the nutrient-related signaling pathway leading to mTOR-mediated induction of autophagy. We measured the degree of phosphorylation of p70S6K, and the levels of both p70S6K and its phosphor-epitope (Thr389n) were tested by western blotting. However, the phosphorylation degree of p70S6K in the hippocampal tissues of 13-month-old APP/PS1 mice was not significant different from that of mice in the WT group, suggesting that autophagy in the brains of mice at this age was not significantly induced. If not, autophagosome degradation may be blocked. Therefore, we tested the autophagy flux via the use of the dynamic fluorescent reporter, mRFP-GFP-LC3, delivered by AAV9. The results indicated that the AVs accumulating in the APP/PS1 mice were mainly autolysosomes, suggesting that the abundant lysosomes are available to fuse with AVs, but the degradation of autolysosomes was blocked. DNLA could significantly reduce the number of AVs in the hippocampus and promoted the degradation of autolysosomes, suggesting that DNLA could improve the function of lysosomal proteolysis. Thus, we further examined the protein expression of cat D, which is the major aspartic protease of lysosomes. The results indicated that mature cat D was impaired in the hippocampi of 13-month-old APP/PS1 mice, and that DNLA could promote the maturation of cat D and enhance the proteolytic activity of cat D.

No endogenous inhibitors of cat D are known and the major factor in regulating mature cat D activity seems to be the pH (Stoka et al., 2016). The damage of Cat D mature in the hippocampi of 13-month-old APP/PS1 mice indicated that the acidic environment in lysosomes may have been alkaline; thus, the acidification degree of lysosomes was further evaluated. We found that M6PR did not dissociate from cat D and the expression of mature cat D decreased in 13-month-old APP/PS1 mice, suggesting that acidification of lysosomes was disordered. The ATP-dependent proton pump, v-ATPase, ensures low intra-lysosomal pH, which is essential for lysosomal hydrolase activity (Mauvezin et al., 2015). v-ATPase is a multi-subunit enzyme composed of a membrane-bound V0 proton pore sector and a cytosolic catalytic V1 sector (Mauvezin et al., 2015). v-ATPase activity is regulated mainly by reversible binding of V1 and V0. The combination of V1 and V0 together plays a role in proton transport, leading to separation failure. Any subunit defect in the V1 subcomplex may affect the binding of V1–V0 sectors. In this study, we found that the v-ATPase A1 subunit was decreased in the APP/PS1 mice, suggesting that the loss of the v-ATPase subunit affected the binding of V1–V0 sectors, thus affecting the acidic environment of lysosomes. DNLA could increase the expression of v-ATPase A1 and improve lysosome acidification.

The expression of v-ATPase A1 was decreased in the brain of APP/PS1 mice, and the acidification function of lysosomes was impaired, which may be partially related to a PS1 mutation. PS1 holoprotein, a specific ligand of the v-ATPase V0a1 subunit, is required for proper *N*-glycosylation, stability, and targeting to lysosomes (Lee et al., 2010). Cells lacking PS1 or both PS1 and PS2 display even greater elevations of lysosomal pH, proteolysis deficits in autolysosomes, and AD-like AV pathology (Lee et al., 2015). Depletion of v-ATPase subunits leads to a loss of lysosomal acidification, but the Ca^2+^/AERCA-dependent fusion remains active. Lysosomal homeostasis is disrupted due to the continuous autophagosome-lysosome fusion in the absence of lysosomal degradation and recycling, which leads to an expansion of the autolysosomal compartment and the accumulation of AVs and Aβ in AVs (Mauvezin et al., 2015).

## Conclusion

We demonstrated that DNLA improve the learning and memory deficit in APP/PS1 transgenic mice, which may be due to promotion of the degradation of intracellular Aβ by increasing the protein level of v-ATPase A1 and then improving autolysosomal acidification and proteolysis.

## Author Contributions

JN, L-SL, and J-SS participated in research design. JN, L-SJ, YZ, YT, and W-JY conducted the experiments. JN and J-SS performed the data analysis. JN, Y-LL, and J-SS wrote or contributed to the writing of the manuscript.

## Conflict of Interest Statement

The authors declare that the research was conducted in the absence of any commercial or financial relationships that could be construed as a potential conflict of interest.

## Supplementary Material

FIGURE S1 The deposition condition of amlyoid protein in brains of 7 m, 9 m, and 13 m old APP/PS1 mice (50×).
