# Supplementary figures and images for: Dendrobium nobile Lindl. Alkaloids Decreases the Level of Intracellular β-Amyloid by Improving Impaired Autolysosomal Proteolysis in APP/PS1 Mice
Source: Front Pharmacol. 2018 Dec 18;9:1479. doi: 10.3389/fphar.2018.01479 (PMC6305391; doi:10.3389/fphar.2018.01479)

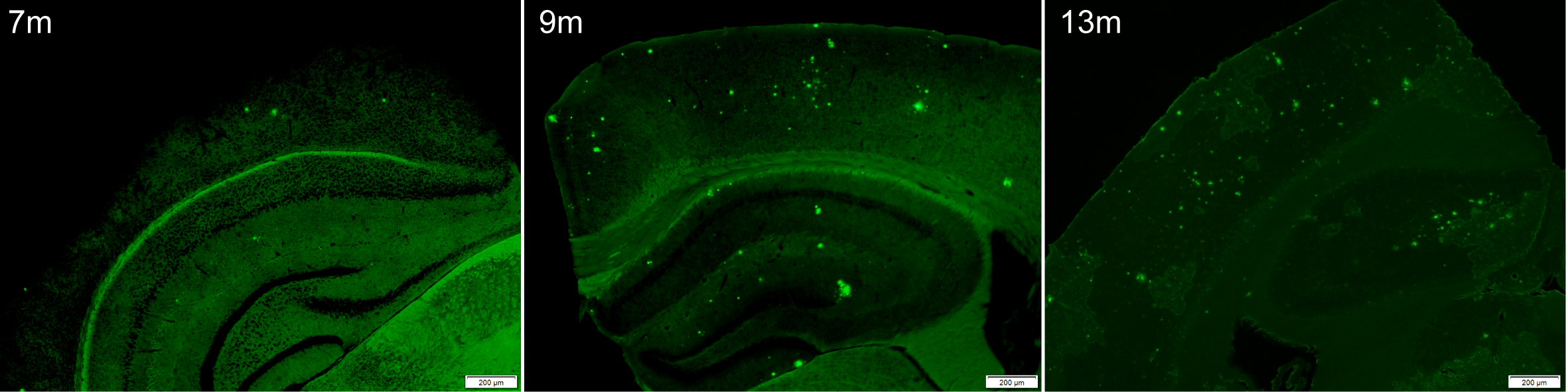

Supplement: FIGURE S1 — The deposition condition of amlyoid protein in brains of 7 m, 9 m, and 13 m old APP/PS1 mice (50×). [file Image_1.TIF]
